# Effects of sociodemographic and socioeconomic factors on stroke development in Lebanese patients with atrial fibrillation: a cross-sectional study

**DOI:** 10.12688/f1000research.54236.1

**Published:** 2021-08-11

**Authors:** Diana Malaeb, Souheil Hallit, Nada Dia, Sarah Cherri, Imad Maatouk, George Nawas, Pascale Salameh, Hassan Hosseini

**Affiliations:** 1 School of pharmacy, Lebanese International University, Beirut, Beirut, Lebanon; 2 Life Sciences and Health Department, Paris-Est University, Paris, France; 3 Faculty of Medicine and Medical Sciences, Holy Spirit University of Kaslik, Jounieh, Lebanon; 4 Institut National de Santé Publique, Epidémiologie Clinique et Toxicologie - Liban (INSPECT-LB), Beirut, Lebanon; 5 Research Department, Psychiatric Hospital of the Cross, Jal Eddib, Lebanon; 6 College of Pharmacy, Xavier University of Louisiana, Louisiana, USA; 7 Faculty of Pharmacy, Lebanese University, Hadat, Lebanon; 8 University of Nicosia Medical School, Nicosia, Cyprus; 9 Life Sciences and Health Department, Paris-Est Créteil University, Paris, France; 10 Henri Mondor Hospital, Paris, France

**Keywords:** Stroke; Lebanon; sociodemographic; CHA2DS2-VASc score

## Abstract

**Background: **Non-communicable diseases, the major cause of death and disability, are susceptible to modifiable and non-modifiable risk factors. Atrial fibrillation (AF) increases the risk of stroke by 4-5 times and can lead to cardiovascular mortality. This study was conducted to assess the effects of different sociodemographic factors on stroke development in patients with AF.

**Methods: **A cross-sectional study was conducted between January and June 2018 on patients recruited from Lebanese community pharmacies. The CHA _2_DS _2_-VASc scoring system is utilized as a stroke risk stratification tool in AF patients. Participants with a previous physician diagnosis of AF, documented on medical records, were included in this study.

**Results: **A total of 524 patients were enrolled in the study with a mean age of 58.75 (± SD) ± 13.59 years with hypertension (78.38%) being the most predominant disease. The results showed that obesity (Beta=0.610, p-value =0.011), retirement and unemployment compared to employment (Beta=1.440 and 1.440, p-value=0.001 respectively), divorced/widow compared to married (Beta=1.380, p-value =0.001) were significantly associated with higher CHA _2_DS _2_-VASc scores whereas high versus low socio-economic status (Beta=-1.030, p=0.009) and high school education versus primary education level (Beta=-0.490, p-value=0.025) were significantly associated with lower CHA _2_DS _2_-VASc scores.

**Conclusions: **The study highlights that the CHA _2_DS _2_-VASc score is affected by the presence of various sociodemographic and socioeconomic characteristics in patients with AF. Thus, screening for those factors may predict the progression of cardiovascular disease and may provide an optimal intervention.

## List of abbreviations

AF Atrial fibrillation

M Mean

NCD Non-communicable diseases

SD Standard deviation

TIA Transient ischemic attack or prior stroke

WHO World health organization

## Introduction

Non-communicable diseases (NCDs), also known as chronic diseases, are responsible for the premature death of 41 million people globally, accounting for 71% of all deaths annually. ^1^ According to the World Health Organization (WHO), ischemic heart disease and stroke are the world’s biggest killers in 2016, accounting for 15.2 million deaths worldwide. ^1^ The global crude death rate per 100,000 population of high-income, upper and lower-middle-income countries due to stroke is the second-highest following ischemic heart disease. ^2, 3^ In Lebanon, NCDs are the major cause of death and disability and are expected to be responsible for 91% of deaths by 2025. ^4, 5^

NCDs are susceptible to modifiable and non-modifiable risk factors that are known to trigger their development. Some behavioral changes can be implemented to halt disease progression as the cessation of nicotine, enhancement in physical activity and adherence to a healthy diet. ^1, 6^ On the other hand, other factors cannot be altered to modify disease development as age, gender, and previous medical conditions.

Atrial fibrillation (AF) increases the risk of stroke by 4-5 times and is estimated to account for ~15% of the 15 million strokes that occur worldwide every year. ^7– 9^ Stroke is expected to increase 2.5 times over the next 40 years due to the increased numbers of older individuals. ^7– 9^ The characteristics of patients with AF in the Gulf and Middle East region is different from the West due to the high prevalence of obesity, diabetes, and smoking in oil-rich countries. ^7^ The risk of stroke development in AF patients is assessed through the CHA _2_DS _2_-VASc model which depends on demographic and clinical risk factors. ^10^ The CHA _2_DS _2_-VASc score allows health care professionals to quickly assess stroke risk and recommend appropriate therapy to prevent further disease progression. ^10^

Sociodemographic and socioeconomic aspects can affect cardiovascular outcomes as certain ethnic groups (African-American/Black), low-income status, low educational level, and unemployment are recognized to negatively impact cardiovascular health. ^11, 12^ In Lebanon, 15% of the population live below the poverty line and 54% in the moderate middle class in 2013. In addition, 54.3% of the Lebanese population aged above 25 years had a secondary level of education in 2017, and the unemployment rate was reported to be 6.2% in 2018. ^13^

There is strong evidence that elucidates that socioeconomic determinants are factors that affect cardiovascular diseases indicating an inverse relationship between socioeconomic status and the occurrence of cardiac diseases. Initially, cardiovascular risk factors and diseases were higher in populations with higher socioeconomic status, but then there was a gradual change where the risk factors were higher in groups with lower socioeconomic status groups. ^14^

Further studies have shown that higher socioeconomic status patients with non-communicable diseases have access to various medical treatments and have greater accessibility to more specialized hospitals with better-prescribed medicines in comparison to the lower socioeconomic status. ^15^

In addition to the socioeconomic status, it has been indicated that structural aspects of an individual's social relationships can predict all-cause mortality from cardiovascular diseases. It has been highlighted that all of the various unmarried states manifested as being single never married, being separated/divorced and being widowed have been all associated with elevated mortality risk from cardiovascular events. ^16^ As far as we know, few studies have been conducted on Lebanese cardiac patients, such as assessing the level of knowledge on warfarin and assessing the impact of pharmacist counseling, but no study has been conducted that evaluated the impact of sociodemographic factors on cardiac disease progression. ^17^

Although the CHA _2_DS _2_-VASc scoring system has been widely adopted as a stroke risk stratification tool in AF patients, it does not take into consideration several of the socioeconomic and sociodemographic factors that contribute to poor cardiovascular outcomes. Accordingly, this study was designed to assess the effect of sociodemographic and socioeconomic factors on CHA _2_DS _2_-VASc score on Lebanese patients with AF.

## Methods

### Study design and setting

A cross-sectional study was conducted on patients recruited from different Lebanese community pharmacies distributed in different Lebanese geographic districts between January and June 2018. The pharmacies were chosen by simple randomization from the list of pharmacies provided by the Lebanese Order of Pharmacists. The questionnaire was administered face-to-face by trained researchers, who had training before the start of the data collection to ensure the quality of research. All participants who presented to the community pharmacies were screened for possible enrollment in the study and included if they had a previous physician-diagnosed AF documented on their electronic medical profile available in the community pharmacies. No attempt was made to verify the accuracy of the physician’s diagnosis. Excluded from the analysis were patients who could not answer the questionnaire adequately either due to a decreased mental alertness or a decreased cognitive function (cognitive disorders, sedated patients, Alzheimer’s disease, etc.). A copy of the questionnaire can be found in the *Extended data.*

### Data collection

The first part of the questionnaire aimed at collecting the patient’s demographic data such as age, gender, work status (employed, retired, unemployed), socioeconomic status (low, moderate, high) and education (primary, middle or high school). The second part included a detailed assessment of the patient’s factors to assess the CHA _2_DS _2_-VASc score. The score calculation is performed on all enrolled patients through an assessment of the following criteria: patients aged ≥65 years, female gender, heart failure, prior stroke or transient ischemic stroke (TIA), hypertension, diabetes mellitus, vascular disease, coronary heart disease or peripheral artery disease. ^18^ All medical diseases were assessed by checking the detailed previous medical conditions along with the prescribed medications documented on medical profiles. These factors were incorporated into a CHA _2_DS _2_-VASc model where each element received 1 point except those aged ≥75 years and those who had a history of stroke or TIA who received 2 points. The CHA _2_DS _2_-VASc score can range from 0 to 9. The score’s interpretation is categorized into “low” if the score is zero, “low-moderate” if the score is one, and “moderate-high” if the score ≥2. ^19^ Some of the questions under the sociodemographic characteristics (as socioeconomic status, educational level, and social history) in the questionnaire were left unanswered either because the patient did not remember or did not understand what was asked. Sources of bias were addressed by minimizing investigators interference in the data collection, patients were recruited in a way to represent the different Lebanese geographic characteristics, and information related to medical history was retrieved from the electronic medical profiles.

### Sample size calculation

Based on a 70% enrolled cardiac patients who were illiterate ^20^ and in the absence of similar studies in Lebanon, the minimal sample size calculated according to the Epi Info software version 7.2 (population survey) was 323 participants to ensure a confidence level of 95%.

### Statistical analysis

An independent person who was unaware of the goals of this research conducted the data coding and analysis using the version 23.0 of the Statistical Package for Social Sciences (SPSS) software. Continuous variables were presented as mean (M) and standard deviation (SD), and the categorical variables were presented as frequency (percentage). The statistical analysis adjusted for the missing values by accounting for the valid percentage in the results output. The Student *t*-test was used to compare the means of the maximum two groups; for means of three and above, the ANOVA test was applied. In the case of non-homogeneity, non-parametric tests substituted the parametric tests. Variables with a p < 0.05 in the bivariate analysis were included in the linear regression to assess its association with the dependent variable, the CHA _2_DS _2_-VASc score. Potential confounders have been eliminated when p > 0.2 to avoid misleading results. P < 0.05 was considered statistically significant.

### Ethics approval and consent to participate

The methods were performed in accordance with the latest relevant guidelines and regulations. The study design was approved by the ethics committee at the Lebanese International University. Written informed consent was obtained from all enrolled patients before the start of the study and their personal identifying information was removed from the data set to respect their autonomy and confidentiality.

## Results

### Sociodemographic characteristics

Out of the 800 questionnaires distributed, 524 patients (65.5%) had AF and were consequently enrolled in the study. Lack of diagnosis by an AF physician is a potential factor for non-enrollment. The sociodemographic and socioeconomic factors of patients included in this study are summarized in Table 1. The mean age of the participants was 58.75 ± 13.59 years, with the majority being males (61.15%) and from rural areas (63.22%). Around 57.2% attended at least high school and 42.02% had a medium economic status. The majority of the studied population (75.6%) had a CHA _2_DS _2-_VASc score of ≥2. As for the past medical history, hypertension (78.38%) and dyslipidemia (67.57%) were the most prevalent concomitant conditions. The mean score (± standard deviation) of the CHA _2_DS _2-_VASc score was 3.07 ± 1.92; out of 524, 206 (39.3%) had a CHA _2_DS _2-_VASc score that is at least equal to the mean.

**Table 1.** Socio-demographic characteristics and past medical history of participants.

Characteristics (N = 524)		Frequency (%) or Mean ± SD
**Age**		58.75 ± 13.59
**Gender**	Male	318 (60.69)
	Female	202 (38.55)
	Missing	4 (0.76)
**Marital status**	Married	426 (81.30)
	Single	38 (7.25)
	Divorced or widow	54 (10.31)
	Missing	6 (1.14)
**Work status**	Employed	252 (48.10)
	Unemployed	158 (30.15)
	Retired	90 (17.18)
	Missing	24 (4.57)
**Dwelling region**	Urban	192 (36.64)
	Rural	330 (62.98)
	Missing	2 (0.38)
**Socio-economic status**	Low	26 (4.96)
	Medium	216 (41.22)
	High	272 (51.90)
	Missing	10 (1.92)
**Education (years)**	Primary school (1-6)	128 (24.9)
	Middle school (7-9)	92 (17.9)
	High school (minimum 10)	294 (57.2)
	Missing	10 (1.92)
**BMI (kg/m** ^**2**^ **)**	Normal (18.5-24.9)	102 (19.47)
	Overweight (25-29.9)	210 (40.77)
	Obese (≥30)	172 (32.82)
	Missing	40 (6.94)
**Current smoking status**	Non- smoker	254 (48.47)
	Smoker	262 (50)
	Missing	8 (1.53)
**Past medical history**	Congestive heart failure	84 (16.54)
	Depression	92 (18.04)
	Deep vein thrombosis or pulmonary embolism	90 (17.72)
	Diabetes mellitus	206 (40.08)
	Hyperlipidemia	350 (67.57)
	Hypertension	406 (78.38)
	Migraine	70 (13.67)
	Peripheral artery disease	70 (13.62)
	Stroke	122 (23.74)
	Transient ischemic attack	40 (7.91)
**Family history**	Heart disease	374 (72.48)
	Stroke	234 (45.88)

%, percentage; SD, standard deviation; BMI, body mass index.

### Bivariate analysis

The results of the bivariate analysis showed a significantly higher CHA _2_DS _2_-VASc score in case of divorce or death of a spouse (p = 0.001). A higher mean score was found in cases of unemployment or retirement compared to employment (p = 0.001). A lower mean score was significantly associated with a higher socioeconomic status (p = 0.001) as well as higher education (p = 0.001). Obesity was significantly associated with a high mean score (p = 0.006) ( Table 2).

**Table 2.** Bivariate analysis of the participants’ characteristics.

Characteristics (N = 524)		Mean ± SD	p-value
**Marital status**	Married	2.95 ± 1.9	0.001
	Single	2.32 ± 1.86	
	Divorced or widow	5.07 ± 2.21	
**Work status**	Employed	2.28 ± 1.68	0.001
	Unemployed	4.08 ± 1.88	
	Retired	4.04 ± 2.1	
**Socio-economic status**	Low	3.92 ± 2.17	0.001
	Medium	3.55 ± 2.13	
	High	2.75 ± 1.87	
**Education (years)**	Primary school (1-6)	4.03 ± 2.08	0.001
	Middle school (7-9)	3.65 ± 2.16	
	High school (minimum 10)	2.59 ± 1.8	
**BMI (kg/m** ^**2**^ **)**	Normal (18.5-24.9)	3.08 ± 2.05	0.006
	Overweight (25-29.9)	2.87 ± 1.97	
	Obese (≥30)	3.53 ± 2.1	
**Residence**	Urban	3.23 ± 2.23	0.387
	Rural	3.07 ± 1.92	
**Current smoking status**	Non- smoker	3.22 ± 2.03	0.423
	Smoker	3.08 ± 2.05	

SD, standard deviation; BMI, body mass index.

### Multivariable analysis

The results of the linear regression showed that obesity (Beta = 0.610, p = 0.011), retirement and unemployment compared to employment (Beta = 1.440, p = 0.001 each), divorced/widow compared to married (Beta = 1.380, p = 0.001), were significantly associated with higher CHA _2_DS _2_-VASc scores whereas high versus low socio-economic status (Beta = −1.030, p = 0.009) and high school education versus primary education level (Beta = −0.490, p = 0.025) were significantly associated with lower CHA _2_DS _2_-VASc scores ( Table 3).

**Table 3.** Multivariable analysis: linear regression taking the CHA _2_DS _2_-VASC score as the dependent variable.

Characteristics (N = 524)		Unstandardized β	Standardized β	p-value	95% CI
**Marital status** (Married ^†^)	Single	−0.220	−0.030	0.507	−0.86; 0.42
	Divorced or widow	1.380	0.210	0.001	0.81; 1.94
**Work status** (Employed ^†^)	Unemployed	1.440	0.330	0.001	1.04; 1.83
	Retired	1.440	0.270	0.001	0.97; 1.92
**Socio-economic status** (Low ^†^)	Medium	−0.660	−0.160	0.089	−1.42; 0.1
	High	−1.030	−0.250	0.009	−1.79; -0.26
**Education (years)** (Primary school ^†^)	Middle school	−0.140	−0.030	0.599	−0.67; 0.39
	High school	−0.490	−0.120	0.025	−0.93; -0.06
**BMI (kg/m**^**2**^**)** (Normal ^†^)	Overweight	0.030	0.010	0.895	−0.43; 0.49
	Obese	0.610	0.140	0.011	0.14; 1.07

^†^ Reference. β, beta; 95% CI, 95 percent confidence interval; BMI, body mass index.

## Discussion

This cross-sectional study was conducted to investigate the impact of sociodemographic and socioeconomic characteristics on CHA _2_DS _2_-VASc score in Lebanese AF patients. Sociodemographic characteristics may directly affect the development of cardiovascular disease that are not currently accounted for in the CHA _2_DS _2_-VASc score criteria. The findings of our study show that obesity, retirement and unemployment compared to employment, and divorced/widow compared to married were significantly associated with higher CHA _2_DS _2_-VASc scores whereas high versus low socio-economic status and high school education versus primary education level were significantly associated with lower CHA _2_DS _2_-VASc scores.

### Effect of job status on CHA _2_DS _2_-VASc score

Our results showed that both jobless and retired participants were associated with higher CHA _2_DS _2_-VASc scores compared to patients with jobs, which is consistent with the results of another study. ^21, 22^ The factors responsible for the increased risk amongst the unemployed are of various causes, which can involve behavioral and lifestyle risk factors, such as smoking, poor diet, alcohol consumption and lack of exercise, all of which increase the risk of cardiovascular disease development and complications. ^23^ In addition to increased smoking and alcohol consumption by unemployed patients, there are other causes, such as financial insecurity and adverse socioeconomic status, which lead to inappropriate health behaviors. Unemployed individuals may not have adequate financial support and/or decreased social involvement which increases the risk of developing cardiovascular and psychological diseases. It has been described that there is a strong correlation between unemployment and the negative effects on cardiovascular and psychological morbidity due to the activation of certain biological factors that are closely linked with acute cardiac events and depression. ^24^

In addition, jobless participants were found to have a higher risk of obesity, lower fruit and vegetable consumption, and restricted physical activity. ^25, 26^ A cross-sectional study in a rural Malay population in Malaysia showed being unemployed or a housewife is associated with metabolic syndrome. ^27^

### Effect of socioeconomic status on CHA _2_DS _2_-VASc score

Our study showed that lower socioeconomic status is associated with higher CHA _2_DS _2_-VASc score which is consistent with the results of another study. ^28^ It is explained that lower socioeconomic status is usually correlated with poor health as the socioeconomic status reflects the level of education, income, and type of occupation. According to a population-based study of 11,247 Australian adults aged ≥25 years, and women with lower education were associated with a higher risk of metabolic disorders manifested by hyperinsulinemia, hypertriglyceridemia, abdominal obesity and hypertension that trigger the risk of cardiovascular occurrence. ^29^ According to the literature, a low educational level is associated with poor health because of the limited access, and lack of comprehensive understanding of health-related information. ^26^ Furthermore, lower socioeconomic factors are not only associated with higher metabolic disorders but are correlated with engagement in a variety of behaviors that exert a detrimental and constructive effect on health. ^30– 32^

The previous findings can be explained by the fact that people from the low socioeconomic factors have a lack of motivation and less hope for the future and as a result, they tend to engage with pleasurable activities to help cope with their situations all of which increase the risk of cardiac disorders. ^33– 35^

### Effect of obesity on CHA _2_DS _2_-VASc score

Our results concluded that obese participants have higher CHA _2_DS _2_-VASc scores compared to normal-weight patients which are reflected in previous studies. ^27, 36^ The literature sheds light that the prevalence of diabetes, hypertension, and dyslipidemia increase with obesity which are all known established risk factors for cardiovascular development. ^37^

It is established that obesity influences the pathogenesis of atherosclerosis through the association with metabolic and inflammatory systems. ^38^ The adipose tissue secretes proteins that are known to regulate biological and physiological states and play a vital role in obesity, atherosclerosis pathogenesis, metabolic derangements, and inflammatory process. ^39^

### Effect of marital status on CHA _2_DS _2_-VASc score

Our results showed that widowed and divorced status were significantly associated with higher CHA _2_DS _2_-VASc score which is consistent with the results of a recent meta-analysis. ^40^ There are several mechanisms postulated to explain the protective effect of marital status on cardiovascular diseases. Social causation theory suggests that individuals benefit from spousal support ^41^ where living with another person, specifically the partner, allows for earlier recognition and response to warning symptoms. ^42, 43^ It was further discussed that single patients had longer delays in seeking medical consultation, ^42, 44, 45^ were more likely to be non-adherent to medications. ^46^ In addition, partners exert a positive effect on the quality of life depicted through the engagement of health behaviors such as a healthy lifestyle and greater financial resources, especially in households with a dual-income making better healthcare more accessible. ^42, 47, 48^ Another reason that explains the effect of marriage on cardiovascular diseases is the stress-related theory, which suggests that partner loss or poor-quality relationships may have a detrimental impact on the economic, behavioral and emotional well-being of an individual that increases susceptibility to diseases. ^49^ Specifically, biological stress may ultimately worsen cardiovascular risk factors such as hypertension, impaired vagal tone, hyperlipidemia, diabetes, and the progression of atherosclerosis. ^50^ It is further highlighted that married status can exert a buffering role through the informational or emotional resources from a spouse promoting adaptive behavior and reducing the excessive neuroendocrine response to acute or chronic stressors translated to a reduced risk of cardiovascular diseases. ^51^

## Limitations

Our study has some limitations: first, the questionnaire used was not validated and self-applied based on subjective assessment of the respondent, so the information could be biased given that recall bias might influence individuals’ responses. Although the patients were recruited from different Lebanese geographic areas yet they might not be representative of the whole Lebanese areas which can hinder generalizing the results to the entire population. In addition, the refusal of some patients to participate in the study might have led to unintended selection bias. Our study has elucidated a preliminary possible association between sociodemographic factors and risk of stroke development among AF patients in Lebanese patients where some risk factors might be population-specific since disease profiles may be different in terms of lifestyle, genetics and demography. A better planned longitudinal study may be of value since the study is cross-sectional where it is impossible to know which comes first, the illness or the socioeconomic factor. Finally, there was no control group. The strengths of our study were the enrollment of a large sample of subjects and the utilization of the validated tool CHA _2_DS _2_-VASc score to assess the odds of stroke in patients with AF.

## Conclusion

This study highlighted the fact that non-communicable diseases should not be assessed through the past or present medical history only; it should rather be evaluated through a thorough investigation of socioeconomic and sociodemographic factors. The findings of this study support the fact that work, economic, and marital status may modify the odds of non-communicable disease in patients with baseline cardiac disease. Consequently, the study raises the need for the integral role pharmacists have in educating and minimizing the influence of socioeconomic factors other than those included in the current guidelines and which can increase the risk for developing cardiovascular disease.

## Data availability

### Underlying data

OSF: Effects of Sociodemographic and Socioeconomic Factors on Stroke Development in Lebanese Patients with Atrial Fibrillation: a cross-sectional study. https://doi.org/10.17605/OSF.IO/PJC6A. ^52^

This project contains the following underlying data.

- Data_Anonymous_Results_stroke with Afib_Dr Diana Malaeb_2020.sav

### Extended data

OSF: Effects of Sociodemographic and Socioeconomic Factors on Stroke Development in Lebanese Patients with Atrial Fibrillation: a cross-sectional study. https://doi.org/10.17605/OSF.IO/PJC6A. ^52^

This project contains the following extended data.

- Questionnaire_Stroke with Afib_Dr Diana Malaeb_2020.doc
- Formulas used in the analysis_Stroke with Afib_Dr Diana Malaeb_2020.docx
- Data key_CV score_Dr Diana Malaeb_2020.docx

Data are available under the terms of the Creative Commons Attribution 4.0 International license (CC-BY 4.0).
