# Supplementary material for: Effects of sociodemographic and socioeconomic factors on stroke development in Lebanese patients with atrial fibrillation: a cross-sectional study
Source: F1000Res. 2021 Nov 10;10:793. Originally published 2021 Aug 11. [Version 2] doi: 10.12688/f1000research.54236.2 (PMC8383125; doi:10.12688/f1000research.54236.2)
Supplement: Peer review history

# Peer review: Reviewer response for version 1

**DOI:** 10.5256/f1000research.57699.r92214

**Published:** 2021-08-26

**Authors:** Muna Barakat

**Affiliations:** 1 Faculty of Pharmacy, Applied Science Private University, Amman, Jordan

Thank you for giving me this opportunity in reviewing the article ”Effects of sociodemographic and socioeconomic factors on stroke development in Lebanese patients with atrial fibrillation: a cross-sectional study”. The article is really interesting and informative as it highlights a vital problem in the community. The findings thus should be used as a tool to enhance patient counselling about the risk factors including diseases and life style measures that should be implemented to minimize the occurrence of chronic diseases. 

 No further comments were raised, and I believe it is suitable to index.

Is the work clearly and accurately presented and does it cite the current literature?

Yes

If applicable, is the statistical analysis and its interpretation appropriate?

Yes

Are all the source data underlying the results available to ensure full reproducibility?

Yes

Is the study design appropriate and is the work technically sound?

Yes

Are the conclusions drawn adequately supported by the results?

Yes

Are sufficient details of methods and analysis provided to allow replication by others?

Yes

Reviewer Expertise:

Clinical practice

I confirm that I have read this submission and believe that I have an appropriate level of expertise to confirm that it is of an acceptable scientific standard.

# Response

**Published:** 2021-09-08

**Authors:** Nada Dia

**Affiliations:** lebanese international university, Lebanon

Dear Dr. Barakat,

 Thank you so much for your time and input.

 The co-authors and I were happy to read your feedback.

 Sincerely,

 Nada Dia and the co-authors

# Peer review: Reviewer response for version 1

**DOI:** 10.5256/f1000research.57699.r92213

**Published:** 2021-08-23

**Authors:** Seif El-Hadidi

**Affiliations:** 1 Faculty of Pharmacy, Future University in Egypt, New Cairo, Egypt

- This article is of good quality, fit for indexing and citation. 

 - The article is well-written and raises an interesting question. 

 - However, I have some weak points for revision before final indexing.

1. Some citations can be added as inserted below. 
  - 1a) Sabry *et al.* (2021 ^1^): This paper is nearly of similar content regarding the social determinants but in COVID-19 among the population of a low-middle Income country. 
  - 1b) PIPHFrEF is a very good statement for meds that are harmful in cardiac diseases & particularly, HF where AF is a prognostic factor (El Hadidi *et al.*, 2020 ^2^).
  - 1c) The role of pharmacists in NCD management might not be well elaborated in the Middle East. El Hadidi *et al.* (2020 ^3^) might be a good reference for pharmacists' role in NCD in limited resources. 
2. The real questionnaire should be attached as supplement material (even if in Arabic/French Language) for reproducibility. 
3. I prefer to change the term "jobless" to "unemployed" as I think it is more ethical.  
4. I prefer to briefly define the criteria/criterion/reference of high/low socioeconomic levels in USD.
5. Some English writings in the intro need to be revised before submission e.g. sentences should not start with abbreviations.
6. In the 4th paragraph of the intro, a very brief comparison of Lebanon people to a neighbouring country might be needed. 

Overall, this manuscript will be very interesting after doing the above recommendations and insertion of the recommended citations.

Is the work clearly and accurately presented and does it cite the current literature?

Yes

If applicable, is the statistical analysis and its interpretation appropriate?

Yes

Are all the source data underlying the results available to ensure full reproducibility?

Yes

Is the study design appropriate and is the work technically sound?

Yes

Are the conclusions drawn adequately supported by the results?

Yes

Are sufficient details of methods and analysis provided to allow replication by others?

Yes

Reviewer Expertise:

Cardiology; Drug-therapy optimisation & Guideline-led Prescribing; Critical Care Medicine; Infectious Diseases; Pharmacoepidemiology.

I confirm that I have read this submission and believe that I have an appropriate level of expertise to confirm that it is of an acceptable scientific standard.

## References

[1] : Awareness of the Egyptian public about COVID-19: what we do and do not know. *Inform Health Soc Care* .2021;46(3) : 10.1080/17538157.2021.1883029 244-255 10.1080/17538157.2021.1883029 33622157

[2] : Potentially Inappropriate Prescriptions in Heart Failure with Reduced Ejection Fraction (PIP-HFrEF). *Eur Heart J Cardiovasc Pharmacother* .2020; 10.1093/ehjcvp/pvaa108 10.1093/ehjcvp/pvaa108 32941594PMC13453067

[3] : Heart Failure Prescribing Quality at Discharge from a Critical Care Unit in Egypt: The Impact of Multidisciplinary Care. *Pharmacy (Basel)* .2020;8(3) : 10.3390/pharmacy8030159 10.3390/pharmacy8030159 32882858PMC7558601

# Response

**Published:** 2021-09-08

**Authors:** Nada Dia

**Affiliations:** lebanese international university, Lebanon

Dear Dr. El-Hadidi,

 Thank you for your time and valuable input.

 Whenever possible, the articles you refer to were added to the section of references. The questionnaire was not attached as it is mentioned in the extended data. A study similar to the one we conducted in Lebanon was hard. Hence comparison was not possible.

 The remaining changes were applied as requested.

 Kindly let us know if further questions/improvements were needed.

 Sincerely,

 Nada dia and the co-authors

# Peer review: Reviewer response for version 1

**DOI:** 10.5256/f1000research.57699.r92211

**Published:** 2021-08-23

**Authors:** Feras Jassim Jirjees

**Affiliations:** 1 College of Pharmacy, University of Sharjah, Sharjah, United Arab Emirates

The manuscript was written in a scientific manner. There are several important points in this study. There are some corrections, especially in the writing, that I would ask the authors to make.

**Abstract:**

- Introduction: “4-5” should be “4 to 5”.
- Aim: the authors should add “socioeconomic factors” to the aim of the study.
- Results: “with a mean age of 58.75 (± SD) ± 13.59 years” should be written as “with a mean age (± SD) of 58.75 ± 13.59 years”.

**Introduction:**

 Page 3:

- Paragraph 3: “~15%”, it is better as "approximately 15%" (no symbol).
- Paragraph 3: “Gulf and Middle East region”, Gulf countries are part of the Middle East region; therefore, it should be the “Middle East countries”.

Page 4:

 Ethics approval and consent to participate:

- No ethics approval number or code, please add it.

**Results:**

 Page 4, Sociodemographic characteristics:

- Number or percentage should either have one digit after decimal or two digits

Page 5, Table 1:

- Age: add (years).

Page 8, Conclusion:

 “…. the study raises the need for the integral role pharmacists have in educating …” It should be healthcare providers instead of pharmacists alone.

 Finally, in general, please use "socioeconomic" instead of “socio-economic” in all of the manuscript.

Is the work clearly and accurately presented and does it cite the current literature?

Yes

If applicable, is the statistical analysis and its interpretation appropriate?

Yes

Are all the source data underlying the results available to ensure full reproducibility?

Yes

Is the study design appropriate and is the work technically sound?

Yes

Are the conclusions drawn adequately supported by the results?

Yes

Are sufficient details of methods and analysis provided to allow replication by others?

Yes

Reviewer Expertise:

Pharmacotherapeutics, Cardiovascular diseases, Diabetes, and medication adherence

I confirm that I have read this submission and believe that I have an appropriate level of expertise to confirm that it is of an acceptable scientific standard.

# Response

**Published:** 2021-09-08

**Authors:** Nada Dia

**Affiliations:** lebanese international university, Lebanon

Dear Dr. Jirjees,

 Thank you for your time and input. All the requested modifications were applied. Nonetheless, the ethics approval number was not listed because in 2018 the Lebanese International University did not release numbers for any accepted study.

 Kindly let us know if further information is needed.

 Sincerely,

 Nada Dia
